# Mucin-Type *O*-Glycosylation in the *Drosophila* Nervous System

**DOI:** 10.3389/fnana.2021.767126

**Published:** 2021-10-18

**Authors:** Kazuyoshi Itoh, Shoko Nishihara

**Affiliations:** ^1^Glycan & Life Systems Integration Center (GaLSIC), Soka University, Hachioji, Japan; ^2^Department of Biosciences, Graduate School of Science and Engineering, Soka University, Hachioji, Japan

**Keywords:** mucin-type *O*-glycans, mucin-type *O*-glycosylation, T antigen, *Drosophila*, nervous system, neuromuscular junction

## Abstract

Mucin-type *O*-glycosylation, a predominant type of *O*-glycosylation, is an evolutionarily conserved posttranslational modification in animals. Mucin-type *O*-glycans are often found on mucins in the mucous membranes of the digestive tract. These glycan structures are also expressed in other cell types, such as blood cells and nephrocytes, and have crucial physiological functions. Altered expression of mucin-type *O*-glycans is known to be associated with several human disorders, including Tn syndrome and cancer; however, the physiological roles of mucin-type *O*-glycans in the mammalian brain remains largely unknown. The functions of mucin-type *O*-glycans have been studied in the fruit fly, *Drosophila melanogaster*. The basic structures of mucin-type *O*-glycans, including Tn antigen (GalNAcα1-Ser/Thr) and T antigen (Galβ1–3GalNAcα1-Ser/Thr), as well as the glycosyltransferases that synthesize them, are conserved between *Drosophila* and mammals. These mucin-type *O*-glycans are expressed in the *Drosophila* nervous system, including the central nervous system (CNS) and neuromuscular junctions (NMJs). In primary cultured neurons of *Drosophila*, mucin-type *O*-glycans show a characteristic localization pattern in axons. Phenotypic analyses using mutants of glycosyltransferase genes have revealed that mucin-type *O*-glycans are required for CNS development, NMJ morphogenesis, and synaptic functions of NMJs in *Drosophila*. In this review, we describe the roles of mucin-type *O*-glycans in the *Drosophila* nervous system. These findings will provide insight into the functions of mucin-type *O*-glycans in the mammalian brain.

## Introduction

Mucin-type *O*-glycans, one of the major types of *O*-glycan, are evolutionarily conserved in animals. They are generally found on mucins, which are mucus glycoproteins expressed on the mucous membranes of the digestive tract, and are necessary for protecting the gut, for example, from physical damage and bacterial infection. Mucin-type *O*-glycans are conjugated to many proteins other than mucins and have been shown to play crucial roles in various mammalian cell types, such as blood cells (Wang et al., 2012; Kudo et al., 2013), nephrocytes (Fuseya et al., 2020; Stotter et al., 2020), and submandibular cells (Tian et al., 2012). In addition, unusual expression of mucin-type *O*-glycans has been associated with several human disorders, including Tn syndrome (Berger, 1999; Ju and Cummings, 2005), IgA nephropathy (Suzuki et al., 2008; Hiki, 2009), heterotaxy (Fakhro et al., 2011; Boskovski et al., 2013), and cancer (Springer, 1984; Ju et al., 2008; Radhakrishnan et al., 2014). However, the functions of mucin-type *O*-glycans in the mammalian brain remains unclear.

In contrast to mammals, the functions of mucin-type *O*-glycans have been well studied in the *Drosophila* nervous system. The genome of *Drosophila*, which is used as a model organism, is about 60% homologous to that of humans. Notably, about 75% of the genes responsible for human diseases are present in *Drosophila* as orthologs (Ugur et al., 2016). About half of the genes encoding human glycosyltransferases have orthologs in *Drosophila* (Yamamoto-Hino et al., 2015), and those orthologs have been shown to synthesize various glycan structures that are conserved in mammals, including some types of mucin-type *O*-glycan such as Tn antigen (GalNAcα1-Ser/Thr) and T antigen (Galβ1-3GalNAcα1-Ser/Thr; Nishihara, 2020). Here, we summarize our current understanding of the roles of mucin-type *O*-glycans in the *Drosophila* nervous system.

### Biosynthesis of Mucin-Type *O*-Glycans in *Drosophila*

In mammals, there are various structures of mucin-type *O*-glycans, including four common core structures (Bennett et al., 2012). In *Drosophila*, by contrast, there are three main glycan structures of mucin-type *O*-glycans, namely, Tn antigen, T antigen (core 1), and glucuronylated T antigen (GlcAβ1-3Galβ1-3GalNAcα1-Ser/Thr; Figure 2A; Kramerov et al., 1996; Aoki et al., 2008; Breloy et al., 2008). Mass spectrometry analysis revealed that the expression levels of Tn antigen, T antigen, and glucuronylated T antigen account for, respectively, about 20%, 55%, and 10% of *O*-glycans in *Drosophila* embryos (Aoki et al., 2008). In mammals and *Drosophila*, polypeptide *N*-acetylgalactosaminyl-transferases (ppGalNAcTs) synthesize Tn antigen by the addition of an *N*-acetylgalactosamine (GalNAc) to serine (Ser) or threonine (Thr) residues of the core protein (Bennett et al., 2012; Tran and Hagen, 2013). Humans and mice have 20 and 19 ppGalNAcTs, respectively, whereas* Drosophila* has 12 ppGalNAcTs.

After the addition of GalNAc, core 1 β1, 3-galactosyltransferase 1 (C1GalT1) transfers galactose (Gal) to the GalNAc residue in a β1, 3-linkage and synthesizes T antigen both in mammals and in *Drosophila*. Whereas mammalian C1GalT1 requires a molecular chaperone, Cosmc (also known as C1GalT1C1), for its enzymatic activity (Ju and Cummings, 2002), *Drosophila* C1GalT1 (dC1GalT1) does not (Müller et al., 2005). After the synthesis of T antigen, sialylated T antigen (Siaα2-3Galβ1-3GalNAcα1-Ser/Thr) is synthesized in mammals; however, sialylated T antigen has not been identified in *Drosophila* (Schwientek et al., 2007; Aoki et al., 2008). In addition, although one sialyltranseferase (SiaT) has been identified in *Drosophila*, it does not show catalytic activity for the transfer of sialic acid (Sia) to T antigen (Koles et al., 2004). Instead of the synthesis of sialylated T antigen in *Drosophila*, glucuronylated T antigen is produced by β1, 3-glucuronyltransferase-P (dGlcAT-P), which predominantly transfers glucuronic acid (GlcA) to the Gal residue in a β1, 3-linkage (Kim et al., 2003; Breloy et al., 2016; Itoh et al., 2018). Therefore, *Drosophila* glucuronylated T antigen is considered to correspond to mammalian sialylated T antigen because the two glycan structures contain negatively charged monosaccharides, namely, GlcA and Sia.

**Figure 1:**
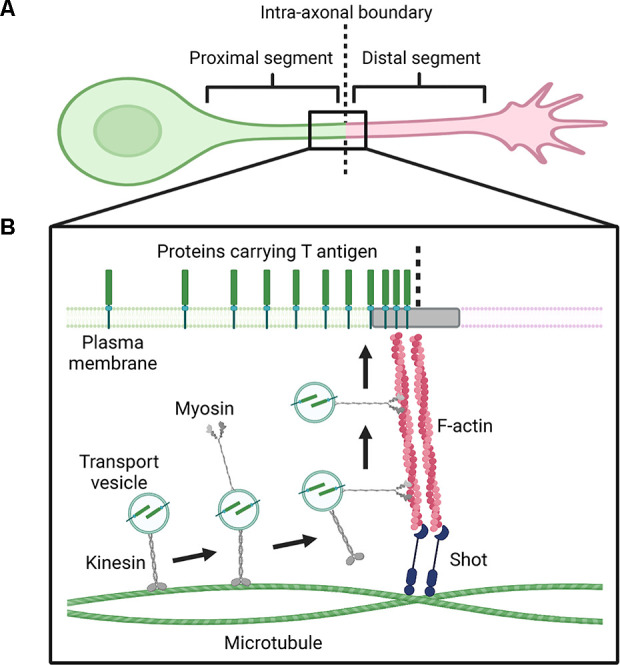
Proposed mechanism of Shot-mediated transport of membrane proteins carrying mucin-type core 1 glycans to the proximal axonal segment. **(A)** Schematic diagram showing a *Drosophila* primary cultured neuron, in which the axon is divided into proximal and distal segments by the intra-axonal boundary. **(B)** Schematic diagram showing the proposed transport mechanism. Transport vesicles containing proteins carrying T antigen are transferred by kinesin on microtubules from the soma to the point where two tubulin bundles are in contact with each other in the axon. The vesicles become detached from the microtubules and are transferred to F-actin, which is linked to microtubules by the crosslinking protein Shot. Subsequently, the vesicles are transferred by myosin on F-actin to the plasma membrane at the intra-axonal boundary, where the membrane proteins carrying T antigen spread to the proximal segment. Figure created with BioRender (https://BioRender.com).

**Figure 2:**
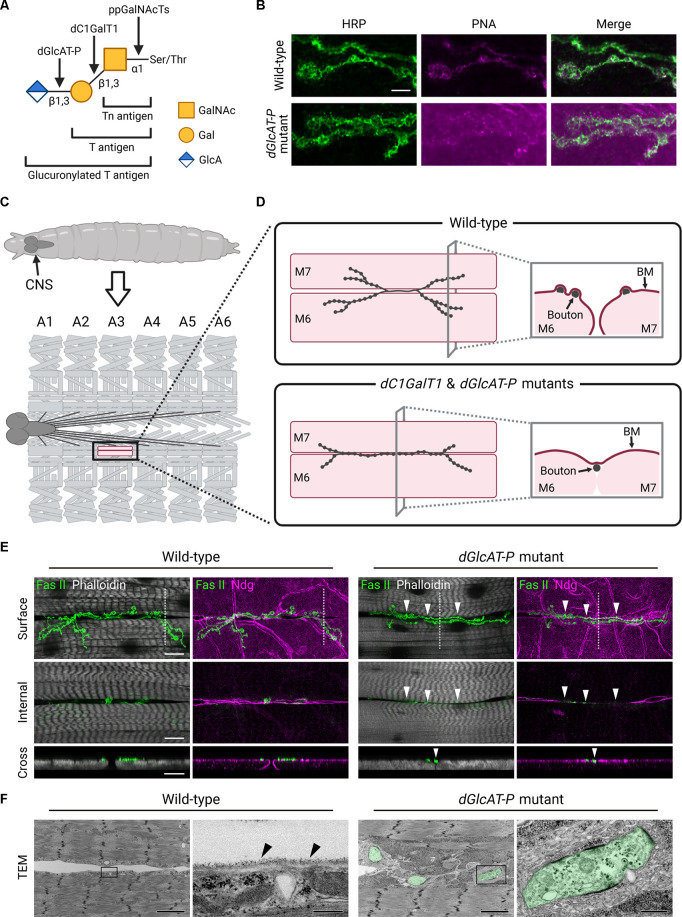
Mislocalization of NMJ boutons and loss of BM components in *dC1GalT1* and *dGlcAT-P* mutants. **(A)** In *Drosophila*, there are three main glycan structures of mucin-type *O*-glycans, namely, Tn antigen (GalNAcα1-Ser/Thr), T antigen (Galβ1-3GalNAcα1-Ser/Thr), and glucuronylated T antigen (GlcAβ1-3Galβ1-3GalNAcα1-Ser/Thr). Polypeptide *N*-acetylgalactosaminyl-transferases (ppGalNAcTs) transfer *N*-acetylgalactosamine (GalNAc) to serine (Ser) or threonine (Thr) residues of the core protein in an α1-linkage to synthesize Tn antigen. Core 1 β1,3-galactosyltransferase 1 (dC1GalT1) transfers galactose (Gal) to the GalNAc residue of Tn antigen in a β1,3-linkage to synthesize T antigen. β1,3-glucuronyltransferase-P (dGlcAT-P) transfers glucuronic acid (GlcA) to the Gal residue of T antigen in a β1,3-linkage to synthesize glucuronylated T antigen. **(B)** Confocal images of larval neuromuscular junctions (NMJs) on muscle 6 in wild-type (upper) and *dGlcAT-P* mutant (lower). NMJs are stained with anti-horseradish peroxidase (HRP) antibody (a presynaptic marker) and peanut agglutinin (PNA; a T antigen marker). T antigen expression colocalizes with the presynaptic marker. The expression level of T antigen at NMJs, as well as on the muscle surface around them, is higher in *dGlcAT-P* mutant than in wild-type. Scale bar: 5 μm. **(C)** Upper, schematic diagram showing *Drosophila* larva. CNS, central nervous system. Lower, schematic diagram showing structures of the nervous system and body wall muscles of a larva, dissected along the dorsal midline. Motor neuron axons extend from the CNS to each abdominal segment, which are numbered in A1–A6. Muscle cells in each hemisegment are regularly arranged, and their arrangement is symmetric. Muscles 6 and 7 in A3 are indicated in magenta. **(D)** Left, whereas NMJ boutons are localized near the boundary between muscles 6 and 7 in wild-type larvae, many NMJ boutons are mislocalized at the muscle 6/7 boundary in *dC1GalT1* and *dGlcAT-P* mutants. The mutant NMJs also show a decreased number of branches. Right, cross-sectional view of muscles 6 and 7. Whereas the two muscles are apart from each other and individually covered with separate basement membranes (BMs) in wild-type, the two muscles are connected through the mislocalized boutons and covered with a single continuous BM in *dC1GalT1* and *dGlcAT-P* mutants. The components of BMs, such as type IV collagen and nidogen (Ndg), are lost *at* or *just beneath* the mislocalized boutons. M6, muscle 6; M7, muscle 7. Figure created with BioRender (https://BioRender.com). **(E)** Confocal images of NMJs on muscles 6 and 7 in A3 in wild-type (left) and *dGlcAT-P* mutant (right). NMJs, muscle fibers, and BMs are stained with anti-fasciclin II (Fas II) antibody, Phalloidin, and anti-Ndg antibody, respectively. Upper, in the surface sectional view of the muscles, NMJ boutons are localized near the boundary between the two muscles in wild-type, but are mislocalized at the boundary in *dGlcAT-P* mutant (arrowheads). Middle, in the internal sectional view of the muscles, each muscle is covered with a continuous BM at the boundary in wild-type, but the BMs are partially lost at the boundary in *dGlcAT-P* mutant (arrowheads). Lower, in the cross-sectional view of the areas within white dotted lines in the upper panels, the two muscles are apart from each other and are individually covered with separate BMs in wild-type, but are attached to each other and covered with a single continuous BM in *dGlcAT-P* mutant. A bouton is ectopically localized at the muscle 6/7 boundary in *dGlcAT-P* mutant (arrowhead). Scale bars: 20 μm (upper and middle panels) and 10 μm (lower panel). **(F)** Transmission electron micrographs (TEM) of the muscle 6/7 boundary in wild-type and *dGlcAT-P* mutant. For each genotype, the right panel shows a high magnification view of the area bordered by the rectangle in the left panel. In wild-type, arrowheads indicate a BM that covers the muscle surface. In *dGlcAT-P* mutant, the presynaptic side of mislocalized NMJ boutons is indicated in green. Whereas no NMJ boutons are observed in the cleft between the two muscles in wild-type, some NMJ boutons are observed in the cleft and connect the two muscles in *dGlcAT-P* mutant. Scale bars: 3 μm (left panels), 300 nm (right panel, wild-type), and 500 nm (right panel, *dGlcAT-P* mutant).

### Expression of Mucin-Type *O*-Glycans in the *Drosophila* Nervous System

#### *In vivo* Expression of Mucin-Type *O*-Glycans

In *Drosophila* embryos, *dC1GalT1* mRNA and T antigen, which can be labeled by anti-T antigen antibody or peanut agglutinin (PNA), are expressed in the central nervous system (CNS), including the brain and ventral nerve cord (VNC; Tian and Ten Hagen, 2007; Lin et al., 2008; Yoshida et al., 2008). T antigen is first expressed in the CNS at embryonic stage 13 and then abundantly expressed in the ladder-like pattern of the VNC at stage 16 (Yoshida et al., 2008). T antigen colocalizes with antigen of the CNS marker BP102. In *dC1GalT1* mutant embryos, T antigen expression in the CNS is completely abolished, showing that dC1GalT1 has a central role in T antigen synthesis in the CNS during embryonic development (Lin et al., 2008; Yoshida et al., 2008).

At the larval stages, *dC1GalT1* mRNA is also detected in the CNS (Lin et al., 2008). Moreover, lectin staining has revealed that both Tn antigen and T antigen are expressed in larval neuromuscular junctions (NMJs; Haines et al., 2007; Dani et al., 2014; Jumbo-Lucioni et al., 2014; Itoh et al., 2016, 2018). In *dC1GalT1* mutant larvae, T antigen expression at NMJs is decreased, showing that dC1GalT1 is required for T antigen synthesis at these synapses (Itoh et al., 2016). Moreover, T antigen expression at NMJs is upregulated in *dGlcAT-P* mutants, suggesting that glucuronylated T antigen is also expressed at these synapses (Figure 2B; Itoh et al., 2018).

Previous studies have revealed that laminin subunits and dystroglycan (Dg), both of which are expressed in the *Drosophila* nervous system, carry mucin-type *O*-glycans (Haines et al., 2007; Bogdanik et al., 2008; Lin et al., 2008; Schneider and Baumgartner, 2008; Nakamura et al., 2010); however, the functions of mucin-type *O*-glycans on these core proteins remain unknown. Dg also carries *O*-mannosyl glycans, which are synthesized by the *Drosophila* protein *O*-mannosyltransferase 1 (dPOMT1) and dPOMT2 (Ichimiya et al., 2004; Ueyama et al., 2010). Loss of either of these two enzymes causes defects in NMJs and axonal connections of sensory neurons (Wairkar et al., 2008; Baker et al., 2018).

#### Localization of Mucin-Type *O*-Glycans in Primary Cultured Neurons

It has been reported that T antigen shows distinctive localization in the axons of primary cultured neurons derived from *Drosophila* embryos (Kinoshita et al., 2017). The axons of primary cultured neurons are divided into two compartments, namely, the proximal segment and the distal segment (Figure 1A; Katsuki et al., 2009). Both the axon guidance receptors, Derailed (DRL) and BP102 antigen are specifically localized to the proximal segment of the axon, while two other axon guidance receptors, roundabout 2 (ROBO2) and ROBO3, are specifically localized to the distal segment of the axon. T antigen localization is also restricted to the proximal segment of axons, similar to DRL and BP102 antigen (Kinoshita et al., 2017).

Ultrastructural analysis by atmospheric scanning electron microscopy (ASEM) has revealed that T antigen accumulates near the boundary between the proximal and distal segments of the axon, known as the intra-axonal boundary. Null mutations of the gene encoding Short stop (Shot), a protein that crosslinks F-actin and microtubules, resulting in impairment of the proximal localization of T antigen, which in turn is rescued by the expression of wild-type Shot. ASEM analysis has revealed that the formation of microtubule bundles is disturbed in Shot null mutants, showing that Shot is involved in microtubule formation. Moreover, loss of the F-actin-binding domain of Shot also results in impairment of the proximal localization of T antigen but does not affect microtubule bundles. Therefore, these results demonstrate that the F-actin-biding domain of Shot is essential for the trafficking of mucin-type core 1 glycans to the proximal axonal segment. ASEM observations have also revealed that F-actin accumulates near the intra-axonal boundary similar to T antigen (Kinoshita et al., 2014). Furthermore, two tubulin bundles in the axon are in contact at the intra-axonal boundary. Taking all these findings together, Kinoshita et al. (2017) have proposed a mechanism for Shot-mediated axonal trafficking of membrane proteins carrying mucin-type core 1 glycans to the proximal axonal segment (Figure 1B).

### Functions of Mucin-Type *O*-Glycans in the Central and Peripheral Nervous System

The phenotypes of the CNS in *dC1GalT1* and *dGlcAT-P* mutants have been analyzed. Although depletion of *dC1GalT1* leads to loss of T antigen expression in the embryonic CNS, the morphology of the CNS is normal (Lin et al., 2008; Yoshida et al., 2008). These data show that T antigen is dispensable for the development of the CNS during embryogenesis. In the larval stages, however, *dC1GalT1* null mutants display a malformed brain hemisphere and greatly extended VNC (Lin et al., 2008), showing that mucin-type core 1 glycans are required for subsequent CNS development. Moreover, this extended VNC phenotype is also observed in *dGlcAT-P* null mutants (Pandey et al., 2011). The *dGlcAT-P* mutant larvae also display a reduction in the length of motor neuron axons that extend from the VNC to target muscles. Because the flies grow extensively during larval stages, motor neuron axons need to extend in parallel to the enlargement of the larval body. Pandey et al. (2011) have therefore suggested that, in *dGlcAT-P* mutants, the VNC extends abnormally in order to compensate for the tension caused by the impaired growth of motor neuron axons. The extended VNC phenotype is rescued by overexpressing *dGlcAT-P* in hemocytes. Collectively, these data suggest the possibility that blood cell-derived mucin-type *O*-glycans produced by dGlcAT-P are involved in the elongation of peripheral nerves.

### Functions of Mucin-Type *O*-Glycans in Neuromuscular Junctions

#### The *Drosophila* Neuromuscular Junction as a Model System for Mammalian Central Synapses

*Drosophila* larval NMJs are a good model for studying synapses in the mammalian CNS (Menon et al., 2013). *Drosophila* NMJs are easy to observe because the synapses are large and specified individually. They are glutamatergic and use ionotropic glutamate receptors (GluRs) that are homologous to AMPA-type GluRs in the mammalian CNS. In *Drosophila* larvae, motor neuron axons stereotypically innervate postsynaptic muscle cells, which are respectively numbered and regularly arranged in each hemisegment (Figure 2C). The NMJs consist of a branched chain of synaptic boutons, which are oval-shaped structures. Each presynaptic bouton is surrounded by subsynaptic reticulum, which is a folded structure of the postsynaptic membrane. The boutons contain an active zone, which is a site of neurotransmitter release, on the presynaptic side and GluR clusters on the postsynaptic side.

#### Phenotypic Analysis of *ppGalNAcT* Mutants

The functions of mucin-type *O*-glycans in NMJs have been studied in *Drosophila* larvae. Loss of either *ppGalNAcT3* or *ppGalNAcT35A* upregulates (i) expression of Tn antigen; (ii) molecular assemblies of presynaptic active zones and postsynaptic GluRs; and (iii) neurotransmission strength evoked at the NMJs (Dani et al., 2014). Ultrastructural observation has shown that the number of presynaptic vesicles near the active zone and the depth of the postsynaptic pocket are increased in the two* ppGalNAcT* mutants. In addition, the components of integrin signaling, including the synaptic Position Specific 2 (αPS2) integrin receptor and transmembrane tenascin ligand, are downregulated at the NMJs of both *ppGalNAcT* mutants. Moreover, the two mutants display the impairment of activity-dependent synaptic plasticity and the suppression of activity-dependent changes in integrin signaling and postsynaptic pocket size. All these phenotypes are restored to wild-type in double mutants of the two *ppGalNAcTs*. Tissue-specific rescue experiments have revealed that, on both the pre- and postsynaptic side of NMJs, *ppGalNAcT3* and *ppGalNAcT35A* regulate Tn antigen expression, synaptic molecular assemblies, neurotransmission strength, and αPS2 integrin expression. Furthermore, inhibition of integrin signaling blocks synaptic plasticity in the two *ppGalNAcT* mutants.

Collectively, therefore, Dani et al. (2014) have suggested that *ppGalNAcT3* and *ppGalNAcT35A* genetically suppress each other on both sides of the synapse to regulate *O*-GalNAc glycosylation, as well as synaptic molecular assemblies, neurotransmission strength, and activity-dependent plasticity through integrin signaling. The suppressive regulation between *ppGalNAcT3* and *ppGalNAcT35A* is apparently linked to the balanced function of the two genes. As a result, the loss of either *ppGalNAcT3* or *ppGalNAcT35A* impairs the balance between the two genes; thus, other *ppGalNAcT* may become dysregulated, leading to the upregulation of Tn antigen expression and other phenotypes as described above. Because of the suppressive interaction between *ppGalNAcT3* and *ppGalNAcT35A*, these phenotypes are restored to wild-type in double mutants.

#### Phenotypic Analysis of *dC1GalT1* and *dGlcAT-P* Mutants

Recent studies have found that mucin-type *O*-glycans synthesized by dC1GalT1 and dGlcAT-P also play crucial roles in larval NMJs. Mutation in either *dC1GalT1* or *dGlcAT-P* leads to morphological defects in the NMJs formed on the large abdominal muscles 6 and 7. In *Drosophila*, the axon terminal of a motor neuron branches and establishes NMJ boutons near the boundary between muscles 6 and 7. While most boutons are localized near the muscle 6/7 boundary in wild-types, many NMJ boutons are ectopically localized at the boundary in *dC1GalT1* and *dGlcAT-P* mutants (Figures 2D,E; Itoh et al., 2016, 2018).

In *Drosophila*, basement membranes (BMs) cover the surfaces of the muscles and NMJ boutons, except for the synaptic cleft between the presynaptic bouton and the postsynaptic muscle (Koper et al., 2012). In the internal sectional view of muscles 6 and 7, wild-type shows continuous BMs at the muscle 6/7 boundary; however, *dC1GalT1* and *dGlcAT-P* mutants display a partial loss of BM components, such as type IV collagen and nidogen (Ndg), at the muscle 6/7 boundary (Figure 2E; Itoh et al., 2016, 2018). In the cross-sectional view of the two muscles, they are individually covered with separate BMs in wild-type; however, in the *dC1GalT1* and *dGlcAT-P* mutants, they are covered with a single continuous BM, and the BM components are lost *at* or *just beneath* the mislocalized bouton at the muscle 6/7 boundary (Figures 2D,E). Further analyses have revealed that the mislocalized boutons in the two mutants tend to localize *at* or *just above* the site of the missing BM components at the muscle 6/7 boundary. Ultrastructural analysis has further revealed that some boutons are localized at the cleft between muscles 6 and 7 and connect these two muscles (Figure 2F). During larval stages, BM components such as type IV collagen are secreted from the fat body to hemolymph (body fluid) and deposited on various tissue surfaces in contact with hemolymph (Pastor-Pareja and Xu, 2011). It is possible that, in the *dC1GalT1* and *dGlcAT-P* mutants, the mislocalized boutons, which connect the two muscles, physically prevent the deposition of BM components just below them, leading to the formation of a single continuous BM across the two muscles. Therefore, the ectopic localization of NMJ boutons at the muscle 6/7 boundary may be a direct cause of the loss of BM components just beneath these boutons. In addition, analysis of double heterozygous mutants of *dC1GalT1* and *dGlcAT-P* reveals that the two genes genetically interact with each other. Taken together, these data clearly demonstrate that glucuronylated T antigen, rather than unmodified T antigen, is essential for the normal localization of NMJ boutons. Moreover, glucuronylated core 1 glycans are also involved in NMJ arborization because *dC1GalT1* and *dGlcAT-P* mutants display a decreased number of NMJ branches on muscles 6 and 7.

Various ultrastructural defects are observed on both the pre- and postsynaptic sides of NMJ boutons in *dC1GalT1* and *dGlcAT-P* mutants (Itoh et al., 2016, 2018). Although most of these defects differ between the two mutants, both show a decrease in the length of postsynaptic density (PSD), which comprises huge protein complexes including GluRs and scaffolding proteins. Therefore, these data suggest that mucin-type *O*-glycans synthesized by dC1GalT1 and dGlcAT-P are also involved in PSD formation.

## Discussion

Although little is known about their function in the mammalian nervous system, the physiological roles of mucin-type *O*-glycans have been well studied in this organ in *Drosophila*. Mucin-type *O*-glycans have been shown to be expressed in the *Drosophila* nervous system, including the CNS and NMJs. In primary cultured neurons, mucin-type core 1 glycans are localized in the proximal axonal segment by Shot-mediated trafficking. Phenotypic analyses have shown that the mucin-type *O*-glycans produced by dC1GalT1 and dGlcAT-P might be involved in CNS development and peripheral nerve elongation. At NMJs, the mucin-type *O*-glycans regulated by ppGalNAcT3 and ppGalNAcT35A control synaptic molecular assemblies, neurotransmission strength, and synaptic plasticity *via* integrin signaling. Moreover, glucuronylated core 1 glycans contribute to normal NMJ bouton localization, NMJ arborization, and PSD formation. Thus, mucin-type *O*-glycans play crucial roles in the *Drosophila* nervous system. Because glucuronylated core 1 glycans are thought to correspond to mammalian sialylated core 1 glycans, as described above, we suggest the possibility that functions may be conserved between these two glycan structures in the nervous system of *Drosophila* and mammals, respectively.

A previous study has revealed that the glycoprotein fasciclin I (Fas I), a homophilic cell adhesion molecule, controls NMJ arborization and synaptic transmission (Zhong and Shanley, 1995), suggesting that cell adhesion between neuron and muscle regulates NMJ morphology and synaptic function. Overexpression of *Fas I* leads to altered morphology of muscle 6/7 NMJs, similar to that observed in *dC1GalT1* and *dGlcAT-P* mutants. In the glomerular epithelium of the mammalian kidney, Podocalyxin carrying negatively charged sialylated mucin-type *O*-gycans has been shown to have anti-adhesive properties, which is required for precise formation of the filtration slit (Takeda et al., 2000; Doyonnas et al., 2001). Negatively charged glucuronylated core 1 glycans in *Drosophila* may also have similar anti-adhesive properties. Therefore, we propose the idea that, in *dC1GalT1* and *dGlcAT-P* mutants, a decrease in glucuronylated core 1 glycans on cell adhesion molecules such as Fas I may facilitate neuron muscle interaction in NMJs and thus cause defects in NMJ arborization and localization.

Previous studies have revealed that ppGalNAcT13 is specifically expressed at a high level in the mouse brain and is essential for the differentiation of neural stem cells through glycosylation and stabilization of podoplanin (Zhang et al., 2003; Xu et al., 2016). Although information about the roles of mucin-type *O*-glycans in the mammalian brain remains limited, recent studies have suggested that mucin-type *O*-glycosylation by several ppGalNAcTs is associated with human brain disorders, including Alzheimer’s disease (Akasaka-Manya et al., 2017; Liu et al., 2017) and a congenital disorder of glycosylation (CDG; Zilmer et al., 2020). Because the basic structures of mucin-type *O*-glycans, including Tn antigen and T antigen, are conserved between *Drosophila* and mammals, the findings in *Drosophila* nervous system will be helpful in our understanding of not only the roles of mucin-type *O*-glycans in the mammalian brain but also the mechanisms underlying human brain disorders.

*Drosophila* is also used as a model system for studying complex brain functions, including cognitive behavior, learning, and sleep. In future studies, analyzing the impact of glycosylation deficiency in these brain functions is likely to lead to the discovery of unprecedented glycan functions in the mammalian brain.

## Author Contributions

All authors listed have edited and revised this manuscript. All authors contributed to the article and approved the submitted version.

## Conflict of Interest

The authors declare that the research was conducted in the absence of any commercial or financial relationships that could be construed as a potential conflict of interest.

## Publisher’s Note

All claims expressed in this article are solely those of the authors and do not necessarily represent those of their affiliated organizations, or those of the publisher, the editors and the reviewers. Any product that may be evaluated in this article, or claim that may be made by its manufacturer, is not guaranteed or endorsed by the publisher.
